# Molecular Identification of Free-Living Amoebae (*Naegleria* spp., *Acanthamoeba* spp. and *Vermamoeba* spp.) Isolated from Un-improved Hot Springs, Guilan Province, Northern Iran

**Published:** 2019

**Authors:** Mohammad Hossein FEIZ HADDAD, Saeed KHOSHNOOD, Mohammad Reza MAHMOUDI, Habib HABIBPOUR, Selman A. ALI, Habibollah MIRZAEI, Rezvan FEIZ HADDAD, Kambiz AHMADIANGALI

**Affiliations:** 1. Infectious and Tropical Diseases Research Center, Health Research Institute, Ahvaz Jundishapur University of Medical Sciences, Ahvaz, Iran; 2. Department of Parasitology, Faculty of Medicine, Ahvaz Jundishapur University of Medical Sciences, Ahvaz, Iran; 3. Student Research Committee, School of Medicine, Bam University of Medical Sciences, Bam, Iran; 4. Molecular and Cellular Research Center, Faculty of Medicine, Guilan University of Medical Sciences, Rasht, Iran; 5. Student Research Committee, Ahvaz Jundishapur University of Medical Sciences, Ahvaz, Iran; 6. Interdisciplinary Biomedical Research Centre, School of Science and Technology, Nottingham Trent University, Nottingham, UK; 7. Hepatitis Research Center, Lorestan University of Medical Sciences, Khorramabad, Iran; 8. Department of Virology, Faculty of Medicine, Ahvaz Jundishapur University of Medical Sciences, Ahvaz, Iran; 9. Department of Midwifery, Faculty of Medicine, Dezful University of Medical Sciences, Dezful, Iran; 10. Department of Bio-Statistics, Faculty of Health, Ahvaz Jundishapur University of Medical Sciences, Ahvaz, Iran

**Keywords:** *Acanthamoeba*, *Naegleria Vermamoeba*, PCR/DNA sequencing, Hot springs, Iran

## Abstract

**Background:**

This study was conducted to determine the presence and molecular identify of *Acanthamoeba*, *Naegleria* and *Vermamoeba* in unimproved hot springs.

**Methods:**

From Jul to Aug 2017, 54 water samples were collected from hot springs in different parts of the Guilan Province, North Iran. For the isolation of *Acanthamoeba*, *Naegleria* and *Vermamoeba* approximately 500 ml of the water samples were filtered through a cellulose nitrate membrane with a pore size of 0.45 μm. The filter was transferred onto non-nutrient agar plates seeded with Gram-negative bacteria (*Escherichia coli*) as a food source. The morphological key of page was used to identify free-living amoebae (FLA) using an inverted microscope, PCR amplification targeting specific genes for each genus and sequencing determined frequent species and genotypes base on NCBI database.

**Results:**

Fifteen of the 54 samples were positive by culture and/or PCR for *Acanthamoeba* and other FLA from unimproved hot springs. By sequencing the positive isolates, the strains were shown to belong to *Acanthamoeba castellanii* (12 case isolates belonged to T4 genotype), 4 cases of *V. vermiformis*, and 3 cases of *N. australiensis*, 2 cases of *N. pagei* and 1 cases of *N. gruberi*.

**Conclusion:**

Although FLA-mediated illnesses are not as high as in environmental distribution, but because of a poor prognosis, more investigations about FLA distribution in hot springs is critical. Hot spring may enhance exposure of the amoebae in individuals. Hence, more attention to unimproved hot springs is needed to prevent free-living amoebae mediated diseases.

## Introduction

Free-living amoebae (FLA) as amphizoic amoebae are a group of parasitic protozoa with the growth abilities in different natural environments such as soil and water. In appropriate conditions, they are pathogenic in animals and humans (1). FLA include many genera which cause serious diseases such as cutaneous ulcers, sight threatening keratitis and fatal encephalitis*.*

*Acanthamoeba* spp., *Naegleria fowleri* and *Balamuthia mandrillaris* are the most commonly reported causes in the world (2). Other genera in this group, including *Sappinia diploidea*, *Vermamoeba* and *Vahlkampfia* mix infection with other FLA could also lead to severe diseases with a lower incidence around the world (3–5). *Acanthamoeba* strains, especially ones that are potentially pathogenic, can tolerate extremes of temperature, osmolarity, and pH (6). *Vahlkampfia* and *Naegleria* genera are commonly found in warm freshwater.

*N. australiensis* and *N. italica* can cause infection in experimental animals but *N. fowleri* is able to infect animals and humans with possible life threatening contamination (7, 8). *Acanthamoeba* spp. *Vahlkammpfids* and *B. mandrillaris* can cause serious infections in humans (1). In addition to *Vermamoeba* have as a suitable hosts for pathogenic microorganisms such as *Legionella pneumophila* (9, 10). Other diseases related to FLA are *Vermamoeba* keratitis, and *Vahlkampfia* keratitis (3, 5, 11).

Hot springs are highly regarded for their therapeutic effects and how much of their use in Iran, both as a therapeutically use and as a tourist attraction, is growing; which will increase the chance of exposure to these amoebae (12–14). Guilan Province is a tourist areas of Iran which plays host to millions of Iranian and foreign travelers annually, and one of the tourist attractions of in this province is its hot springs (15). The presence of FLA in the hot springs of the provinces bordering Guilan has been confirmed (14, 16). Parasitological methods only recognize parasite contamination however, it is not possible to identify the exact species of parasite involved (17).

Hence, we aimed to survey the incidence of waterborne FLA belonging to *Vermamoeba*, *Naegleria* spp. and *Acanthamoeba* spp. isolated from unimproved therapeutic hot springs of Guilan Province using morphological and sequence based methods.

## Materials and Methods

### Geographical area of study

**Fig. 1:**
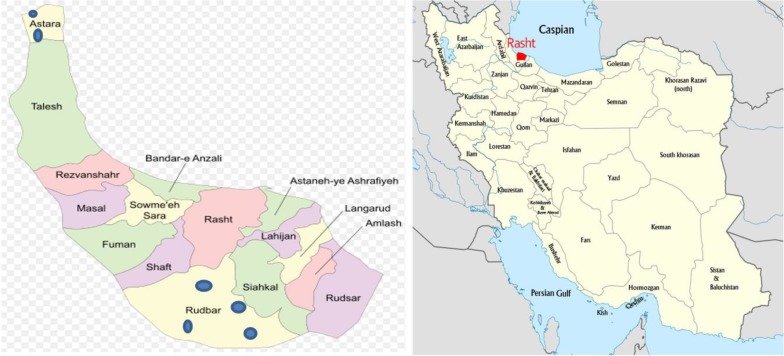
Map of the investigated Guilan greater area (left) and its location in Iran (right) The sampling points are indicated by blue circles (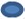)

Guilan Provinces lies along the Caspian Sea. It has a plenty of annual rainfall, humid temperate weather and is known for its mild, moderate and Mediterranean-like climate (15) (Fig. 1).

### Sampling

This cross sectional study (Code of ethics: IR.AJUMS.REC.1396.501) was performed from July to August 2017. To run this study, 54 water samples were collected from hot springs in different parts of the Guilan Province, North Iran (Fig. 1). Six hot springs in Guilan, North of Iran were included in this study and nine samples per site (reservoir- margin- center) were obtained. Samples were collected from the surface of the waters (<5 cm below) (14).

### Filtration, cultivation and cloning

Approximately 500 ml water samples were filtered using cellulose nitrate membranes with a pore size of 0.45 μm. Filters were transferred to non-nutrient agar (NNA) plates seeded with *Escherichia coli* as a food source (14, 15). The morphological key of page was use for the identification FLA using an inverted microscope. Cloning of the suspected amoebae was performed using culture replicates method (11, 18).

### DNA extraction, PCR analysis and Sequencing

DNA was extracted using GeNet Bio kit, according to manufacturer's instructions (GeNet Bio, South Korea). Four sets of primers were used in order to detect various FLA shown in Table 1. To carry out the PCR reaction, 25 ml of red master mix (Denmark) was combined with DNA (10 ng), 0.1 μM of each primer and distilled water. The cycling condition was set as pre-denaturation step for 3 min at 94 °C, followed by 35 repetitions at 94 °C for 35 sec, annealing steps were at 56 °C, 56 °C, 56 °C and 58 °C for 1 min (for *Acanthamoeba, Vahlkampfiids*, *N. fowleri* and *Vermamoeba,* respectively), and 72 °C for 1 min. All sequences were edited manually and analyzed with reference sequences by Chromas software program. The sequences were submitted to gene bank under following accession numbers: MH347242-MH347263.

**Table 1:** Primers used in this study

***FLA^1^***	***Primer sequence***	***Reference***
*Acanthamoeba* spp.	JDP15′-GGCCCAGATCGTTTACCGTGAA-3′	19
	JDP2 5′-TCTCACAAGCTGCTAGGGAGTCA-3′	
*Vahlkampfiids*	ITS1 F5′-GAACCTGCGTAGGGATCATTT- 3′	2
	ITS2 R 5′TTTCTTTTCCTCCCCTTATTA-3′	
*N. fowleri* *^2^*	F5′-GTGAAAACCTTTTTTCCATTTACA-3′	14
	R5′-AAATAAAAGATTGACCATTTGAAA-3′	
*Vermamoeba*	Hv1227F 5′- -TTA CGA GGT CAG GAC ACTGT-3′	2
	Hv1728R 5′-GAC CAT CCG GAG TTC TCG-3′	

1 . Free living amoebae: FLA

2 . *Naegleria fawleri*

## Results

Of 54 water samples, 15 (27.7%) cases were positive for outgrowth of free-living amoebae. Indeed, the positive samples included 7 cases of *Acanthamoeba*, 2 cases of *Vahlkammpfids*, 1 case of *Vermamoeba*, 2 mixed cases of *Acanthamoeba*, *Vahlkammpfids* and *Vermamoeba*, 2 mixed cases of *Acanthamoeba* and *Vahlkammpfids*, and 1 mixed case of *Acanthamoeba* and *Vermamoeba*. Accordingly, *Acanthamoeba* were found in 12 (54.5%) samples as the most prevalent amoebae in the tested samples (Tables 1, 2).

**Table 2:** Data regarding isolated of free-living amoebae in Guilan Hot Springs, and Samples Sites

***City***	***Sampling Site***	***No. of Samples***			***Number/positive samples***	***Acanthamoea***	***Vahlkam pfid***	***Vermamoeba***	***Mixed Acanthamoeba, Vahlkampfiid and Vermamoeba***	***Mixed Acanthamoeba and Vahlkampfi id***	***Mixed Acanthamoe a and Vermamoeba***
		***R***	***C***	***M***							
Roud	Kolour	3	3	3	9/2	1	1	0	0	0	0
bar	Louye	3	3	3	9/4	2	0	0	1	1	0
	Mastkhor	3	3	3	9/4	3	0	0	0	1	0
	Kalashtar	3	3	3	9/2	1	0	0	0	0	1
Astara	Alidashi	3	3	3	9/3	0	1	1	1	0	0
	Koutekoume	3	3	3	9/0	0	0	0	0	0	0
Total	6	1	1	1	54/15(27.7%)	7	2	1	2	2	1
		8	8	8							

R: Reservoir C: Center M: Margin

**Fig. 2:**
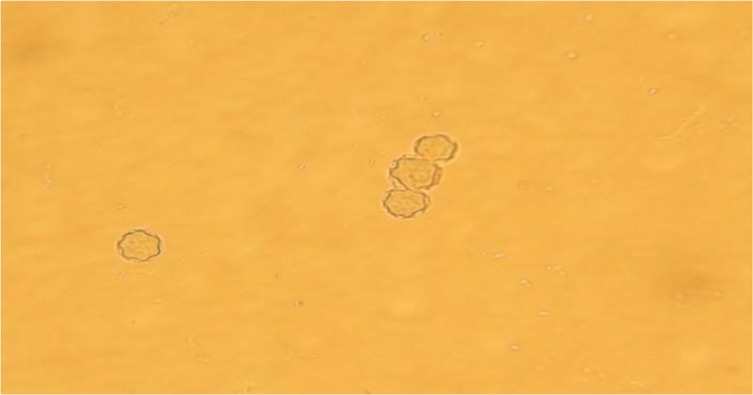
*Acanthamoeba castellanii* cysts (400 X)

**Fig. 3:**
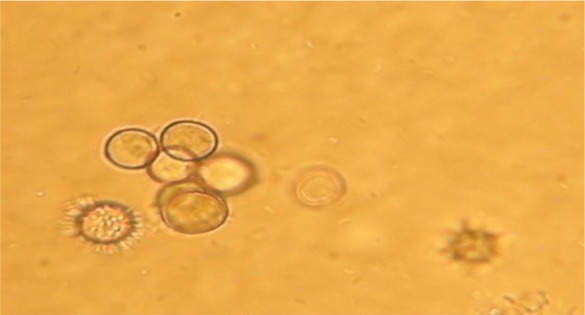
Mixed *Vahlkammpfids* cysts (1) (400 X) and *Acanthamoeba castellanii* trophozoite (2) (400 X)

**Fig. 4:**
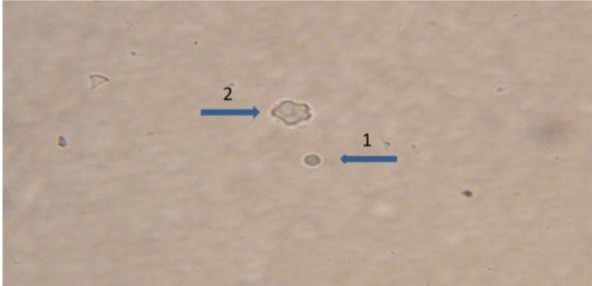
Mixed *Vermamoeba vermiformis* cysts (1) (400 X) and *Acanthamoeba castellanii* cysts (2) (400 X)

For morphological determination of *Acanthamoeba castellanii*, the following characters were regarded; a double walled with a smooth wrinkled outer cyst wall and stellate endocyst (Figs. 2 and 3) for *Vahlkammpfids* round cysts with smooth wall and *V. vermiforims* round cysts with smooth wall but smaller *Vahlkammpfids* (Fig. 3 and 4).

**Fig. 5:**
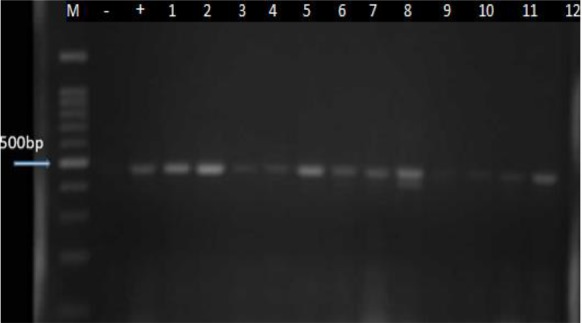
PCR amplification of the isolated *Acanthamoeba* strains. M marker, -=Neg Control, +=Pos Control, 1–12 samples

In electrophoresis of PCR products, twelve cases of *Acanthamoeba* demonstrated an approximately 500 bp band (Fig. 5). Sequencing analysis of 12 positive cases showed T4 (100%) genotypes with homology analysis of NCBI website revealing 95%-100% similarity.

**Fig. 6:**
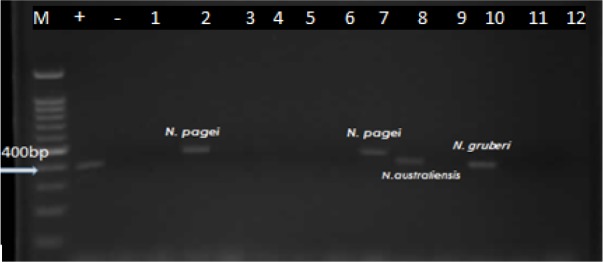
PCR amplification of the isolated *naegleria* strains. M marker, -=Neg Control, +=Pos Control, (2, 7,8 and 10 samples)

**Fig. 7:**
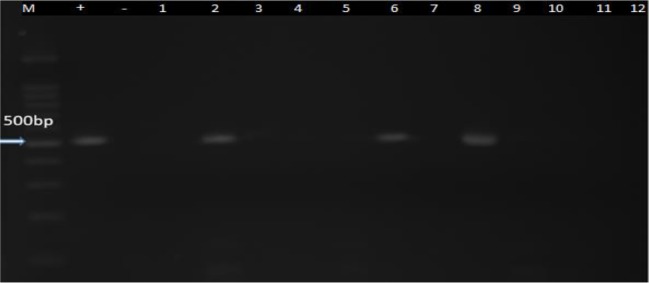
PCR amplification of the isolated *Vermamoeba*. M marker, - = Neg Control, + = Pos Control, (2,6 and 8 samples)

Taken together, 6 cases of *Vahlkammpfids* (2 single and 4 mixed with *Acanthamoeba*) and 4 cases of *Vermamoeba* (1 single and 3 mixed with *Acanthamoeba*) were determined. Six cases of *Vahlkammpfids* exhibited an approximately 400 bp band, and 4 samples of *Vermamoeba* demonstrated a nearly 500bp band during electrophoresis (Figs. 6 and 7).

Water PH and temperature were assessed in situ by using a portable pH meter (Digital tester DMT-20), so that these parameters of hot springs were respectively measured as 23.4%-31.6 °C and 6.5–7.2 (Table 3).

**Table 3:** Data of the free living amoebae from unimproved hot springs, Guilan Province, Iran

***Name Hot Spring***	***Isolate code***	***Morphology***	***PH***	***Temperature °C***	***PCR (JDP1,2)***	***PCR (ITS1, 2)***	***PCR for N. fowleri***	***PCR (Hv1227F, Hv1728R)***	***Sequencing***	***Accession Number***
Kolour	RN2	*Acanthamoeba*	7.1	31.6*	+	-	-	-	T4 genotype	MH347242
	RN3	*Vahlkamfiid*	6.8	31.2	-	+	-	-	*N. pagei*	MH347254
Louye	RN14	*Acanthamoeba*	6.5*	30.3	+	-	-	-	T4 genotype	MH347243
	RN16	*Acanthamoeba*	7	30	+	-	-	-	T4 genotype	MH347244
	RN17	*Acanthamoeba*,*Vahlkamfiid* *Vermamoeba*	6.8	30.5	+	+	-	+	T4 genotype *N. Pagei* *V.vermiformis*	MH347245 MH347255 MH347260
	RN18	*Acanthamoeba*, *Vahlkamfiid*	7.1	23.4*	+	+	-	-	T4 genotype *N. gruberi*	MH347246 MH347256
Mastkhor	RN20	*Acanthamoeba*	6.6	23.6	+	-	-	-	T4 genotype	MH347247
	RN21	*Acanthamoeba*, *Vahlkamfiid*	6.8	23.4	+	+	-	-	T4 genotype *N. australiensis*	MH347248 MH347257
	RN22	*Acanthamoeba*	7.2*	30.6	+	-	-	-	T4 genotype	MH347249
	RN23	*Acanthamoeba*	6.6	30.1	+	-	-	-	T4 genotype	MH347250
Kalashtar	RN30	*Acanthamoeba*	7	28.4	+	-	-	-	T4 genotype	MH347251
	RN36	*Acanthamoeba,Vermamoeba*	6.8	27.6	+	-	-	+	T4 genotype *V.vermiformis*	MH347252 MH347261
Alidashi	AN43	*Acanthamoeba.Vahlkamfiid* *Vermamoeba*	6.6	27.8	+	+	-	+	T4 genotype *N. australiensis* *V.vermiformis*	MH347253 MH347258 MH347262
	AN44	*Vahlkamfiid*	7	30.4	-	+	-	-	*N. australiensis*	MH347259
	AN45	*Vermamoeba*	7.2	28.9	-	-	-	+	*V.vermiformis*	MH347263

* = maximum and minimum water PH and temperature

Moreover, sequencing analysis revealed 95%–100 % similarity with *Vahlkammpfids* and *V. vermiformis*. Accession numbers of nucleotide sequences were deposited in the GenBank database, and have been demonstrated in Table 3. Moreover, six hot springs, 83.4% (n=5), 66.6% (n=4) and 50% (n=3) were respectively positive for *Acanthamoeba*, *Vahlkamfiids*, and *Vermamoeba* amoebae.

## Discussion

The present study is the first study on un-improved hot springs of Guilan Province, Northern Iran to determine the pathogenic free-living amoeba via molecular methods. In this investigation waterborne free living amoebae belonging to the *Acanthamoeba* T4 genotype, *Naegleria* (*N. pagei, N. australiensis* and *N. gruberi*) and *Vermamoeba verformis* were found in the unimproved hot springs of Guilan Province, Northern Iran and the present study is the second report of *N. gruberi* in the country. *Acanthamoeba* was detected in surface water of Guilan, previously (15).

No significant differences were shown between pH value (and temperature) and the presence/absence of *Acanthamoeba*, *Naegleria*, *Vermamoeba*. In previous studies, the T4 genotype was reported to be isolated from samples such as soil, hospital wards, surface waters, recreational water areas, dust sources and also hot springs in Iran (13, 19–23). In contrast with results reported by other studies that reported T15 and T3 as predominate genotypes in waters surveyed (11, 24), the founding of PCR analysis and sequencing in the present work confirmed that the T4 genotype was the predominant type (Table 3).

Among the 47 known species of *Naegleria,* only *N. fowleri* has been reported to be pathogenic for human (8). An investigation on hot springs sources in Iran reported an increased occurrence of *Naegleria* genus in the tested samples (14). The pathogenic *N. fowleri* was not found in this study. To our knowledge, so far, there is no report on the presence of pathogenic *N. fowleri* in environmental sources of Iran.

However, a clinical case of *N. fowleri* has been reported in the country (25).

In the present study, *N. australiensis* was the most prevalent species, which can be pathogenic to mouse (26). *V. vermiforims* was also one of the most detected FLA, based on molecular assays (Table 3). A case of *Vermamoeba* keratitis and a case of mix infection of *V. vermiforims* and *Acanthamoeba* were reported during previous studies (4, 5, 27).

To prevent infection and diseases related to free-living amoebae, hot springs should be periodically checked, in particular, during the summer season, when these surface water are used by thousands of tourists (28). The disease could originate to possess a seasonal mode of frequency in the region and serious monitoring for proper preparation against the disease should be in place (29).

## Conclusion

Although FLA-mediated illnesses is not as high as in their environmental distribution, because of a poor diagnosis, more investigations about FLA distribution in hot springs is critical. Hot spring may enhance exposure of the amoebae to individuals. Hence, more attention to unimproved hot springs is needed to prevent free-living amoebae mediated diseases.
